# Phytometabolite Dehydroleucodine Induces Cell Cycle Arrest, Apoptosis, and DNA Damage in Human Astrocytoma Cells through p73/p53 Regulation

**DOI:** 10.1371/journal.pone.0136527

**Published:** 2015-08-26

**Authors:** Natalia Bailon-Moscoso, Gabriela González-Arévalo, Gabriela Velásquez-Rojas, Omar Malagon, Giovanni Vidari, Alejandro Zentella-Dehesa, Edward A. Ratovitski, Patricia Ostrosky-Wegman

**Affiliations:** 1 Departamento de Medicina Genómica y Toxicología Ambiental, Instituto de Investigaciones Biomédicas, Universidad Nacional Autónoma de México, México, D. F., Mexico; 2 Departamento de Ciencias de la Salud, Universidad Técnica Particular de Loja, Loja, Ecuador; 3 Departamento de Química Aplicada, Universidad Técnica Particular de Loja, Loja, Ecuador; 4 Dipartimento di Chimica Organica, University of Pavia, Pavia, Italy; 5 Departamento de Bioquímica, Instituto Nacional de Ciencias Médicas y Nutrición“Salvador Zubirán”, México, D. F., Mexico; 6 Head and Neck Cancer Research Division, Johns Hopkins University School of Medicine, Baltimore, United States of America; Winship Cancer Institute of Emory University, UNITED STATES

## Abstract

Accumulating evidence supports the idea that secondary metabolites obtained from medicinal plants (phytometabolites) may be important contributors in the development of new chemotherapeutic agents to reduce the occurrence or recurrence of cancer. Our study focused on Dehydroleucodine (DhL), a sesquiterpene found in the provinces of Loja and Zamora-Chinchipe. In this study, we showed that DhL displayed cytostatic and cytotoxic activities on the human cerebral astrocytoma D384 cell line. With lactone isolated from *Gynoxys verrucosa* Wedd, a medicinal plant from Ecuador, we found that DhL induced cell death in D384 cells by triggering cell cycle arrest and inducing apoptosis and DNA damage. We further found that the cell death resulted in the increased expression of CDKN1A and BAX proteins. A marked induction of the levels of total TP73 and phosphorylated TP53, TP73, and **γ**-H2AX proteins was observed in D384 cells exposed to DhL, but no increase in total TP53 levels was detected. Overall these studies demonstrated the marked effect of DhL on the diminished survival of human astrocytoma cells through the induced expression of TP73 and phosphorylation of TP73 and TP53, suggesting their key roles in the tumor cell response to DhL treatment.

## Introduction

Although the development of novel anti-cancer therapeutics has increased over the previous decades, the battle against cancers is far from over. Serious problems associated with the great diversity of human tumors remain, including their clonal nature and origin from adult stem cells, acquired resistance to known chemotherapeutics agents, inability to efficiently eliminate cancer cells without harming the adjacent normal cells, and many others. Thus, the quest for more efficient, specific and natural anti-cancer compounds is still ongoing. Plant-derived active phytometabolites, as well as their semi-synthetic and synthetic analogs, have served as a major route to the development of new pharmaceuticals compounds [1]. Currently, there are more than 200 naturally produced drugs in preclinical/clinical development or in the clinic [2]. The therapeutic properties of medicinal plants are generally attributed to secondary metabolites, such as sesquiterpene lactones, which constitute a large and diverse group of biologically active chemicals that have been identified in several plant families [3]. Sesquiterpene lactones are plant-derived compounds often used in traditional medicine against inflammation and cancer [3]. The greatest numbers of sesquiterpene lactones are found in the Asteraceae family with over 3000 reported structures [4]. One member of the Asteraceae family, the plant *Gynoxys verrucosa* Wedd, which is known as “guángalo” or “congona”, is a shrub grown in the provinces of Loja and Zamora-Chinchipe and has been used by the indigenous population of South America (e.g., Ecuador, Columbia, Peru) for medicinal purposes for ages [5]. Among the secondary metabolites isolated from this species is the sesquiterpene lactone Dehydroleucodine (DhL), which possesses anti-inflammatory, anti-parasitic and anti-microbial activities [6–9]. The main goal of this work was to determine whether DhL may display cytostatic, cytotoxic and genotoxic activities on human cancer cells.

## Materials and Methods

### Extraction of Dehydroleucodine from *Gynoxys verrucosa*

A corresponding research permission of the Ministerio del Ambiente of Ecuador was received to carry out the collection of the plant species *Gynoxys verrucosa* Wedd. It was verified that this species is not endangered and not protected according to “*Libro rojo de las plantas endémicas del Ecuador*” [10]. The materials of *G*. *verrucosa* were collected in 2004 in Yangana, Loja, Ecuador, on a private property with the appropriate authorization of the homeowner. A voucher specimen was deposited into the Herbarium of the Instituto de Química Aplicada de la Universidad Técnica Particular de Loja, Ecuador. The phytobiomass of *G*. *verrucosa* was extracted with methanol at room temperature, which was followed by evaporation of the solvent in a vacuum. Dehydroleucodine was isolated and characterized (S1 Fig), as previously described [6]. Dehydroleucodine stock solutions (1000 μM) were prepared with dimethylsulfoxide (DMSO) 100% and stored at -20°C. The aliquots were diluted to obtain the desired concentrations before use. All chemicals were purchased from Sigma-Aldrich Corporation (St. Louis, MO, U.S.A.)

### Cell Lines

Human astrocytoma D384 cells were a kind gift from Drs. Mayra Paolillo and Uliana de Simone at the University of Pavia. The cells were tested for mycoplasma contamination and authenticated at the tissue bank of the Istituto Zooprofilattico Sperimentale Della Lombardia E’Dell’Emilia Romagna (IZSLER, Brescia, Italy) using an isoenzyme analysis and a PCR RFLP reaction, as well as authenticated by STR-DNA typing. Human kidney clear cell carcinoma Caki-1 (HTB-46) cells, breast cancer MCF-7 (HTB-22) cells, and lung carcinoma A549 (CCL-185) cells were purchased from the American Type Culture Collection (ATCC, Manassas, VA, U.S.A.). D384 cells, Caki-1 cells, MCF-7 cells, and A549 cells were cultured in RPMI-supplemented medium (100 units/mL penicillin G, 100μg/mL streptomycin, 0.25μg/mL amphotericin B) with 2 mM L-glutamine with 10% fetal bovine serum (FBS, v/v, Invitrogen, Carsbad, CA, U.S.A.) in a humidified incubator (37°C, 5% CO_2_). The doubling times of the Caki-1, MCF-7, A549 cells were established as 24 h, while that for D384 cells was 16 h.

### Cell Viability Assay

Cell viability was analyzed using an MTT assay, which is used to assess the viability and/or the metabolic state of the cancer cells based on mitochondrial respiratory activity. A total of 5x10^3^ cells were seeded into each well of 96-well plates and allowed to adhere for 24 h. The cells were then treated with the methanol extract *Gynoxys verrucosa* (50 μg/mL) and DhL (50 μM) for the indicated periods of time. Each concentration/assay was performed in triplicate. Negative control cells were treated with the vehicle DMSO to a final concentration of 0.1% v/v), and positive control cells were treated with Doxorubicin (1 μM) for the indicated time periods.

After incubating the cells with drugs, MTT (5 mg/mL) was added, and the cells were further incubated for 4 h at 37°C. The medium was then aspirated, and the resulting crystals were dissolved in 150μL of DMSO. The absorbance was measured at 570 nm against the reference wavelength of 650 nm. The fraction of inhibition was calculated based on the following formula: viability (%) = absorbance of treated cells/absorbance of control cells x 100%.

### Observation of Cell Morphological Changes

D384 cells were cultured at a density of 1 × 10^6^ cells/mL in 60 mm culture dishes. After 16 h of attachment, the cells were treated without or with various concentrations of DhL for 6 h, 16 h and 32 h. After treatment, the cells were visualized under an inverted phase-contrast microscope (Nikon) to observe and image the cell morphological changes upon treatment.

### Mitotic and Replication Indexes

For mitotic and replication indexes and cell cycle analysis, D384 cells were synchronized. The cells were seeded at 4.8×10^4^ cells/well in a 6-well culture plate containing RPMI (Gibco/Life Technologies, Grand Island, NY, U.S.A) media free of FBS for 16 h to force cells into a quiescent state by depriving the cells of nutrients. The cells were then released from the quiescent state by adding fresh medium containing FBS. The cells were treated with DhL for 16 h during which the medium was also supplemented with BrdU (3.24 μM), and after 32 h of culture, colcemid (0.13 μg/mL) was added to inhibit cytokinesis. Finally, after 24 h, the cultured cells were incubated in a hypotonic solution (0.075 M KCl) for 30 min (37°C, 5% CO_2_) to break the cellular membranes. After fixation (methanol/acetic acid 3:1), the cells were placed onto slides and stained according to the fluorescence plus Giemsa method to differentiate sister chromatids. Microscopic analysis was performed to determine the mitotic index (MI) scoring the number of metaphases in 2000 cells. The proportion of first (M1), second (M2) and third or more (M3) mitotic divisions in 100–200 metaphases was evaluated to determine the replication index (RI) according to the formula RI = (M1+2M2+3M3)/ total analyzed metaphases [11].

### Cell Cycle Analysis

Cell cycle distribution was assessed using propidium iodide (PI) staining. D384 cells were seeded in 60 mm culture dishes (1 × 10^6^ cells) and incubated for 24 h to allow attachment. The cells were then treated with DMSO (0.1% v/v) and DhL (5–10 μM) for 16 h. Only adherent cells were harvested, washed with PBS, and the cell pellets were re-suspended in 100 μL of PBS, fixed with absolute ethanol and stored at −20°C for 24 h. The fixed cells were washed twice with PBS, and the cell pellets were then incubated in a buffer containing 50 μg/mL PI, 0.1% sodium citrate, 0.1% Triton-X-100, and 100 μg/mL RNA-ase A for 30 min in the dark at room temperature. The fraction of cells in the G1, S, and G2/M-phases of the cell cycle was then analyzed using a FACSCanto II flow cytometer (Becton Dickinson, Franklin Lakes, NJ, U.S.A.). The data were acquired and analyzed using DIVA software (Becton Dickinson). Integration of the area under the curve for each of the phases of the histogram was performed with ModFit LT software (Becton Dickinson).

### Clonogenic Assay of Cells In Vitro

The cells were seeded in 100 mm Petri dishes at a low density (1x10^4^ per dish) and left to adhere for 24 h in standard medium. Increasing concentrations of DhL were added; after 16 h, the cells were washed, treated with trypsin, re-suspended in single-cell suspension, and 100 cells were seeded per plate to determine macroscopic colony formation. After 15 days of growth, colonies were fixed with a 3:1 mixture of methanol/acetic acid and stained with crystal violet. Only colonies consisting of more than 50 cells were scored. The experiments were performed in duplicate on three separate occasions. Three separate experiments were performed in duplicate.

### Annexin V-FITC/PI Apoptosis Assay

Flow cytometry analysis was performed to identify and quantify apoptotic cells using an Annexin V-FITC/PI apoptosis detection kit (BD Bioscience). Briefly, the D384 cells were seeded in 6-well plates at a density of 1 x 10^5^ cells/well. After treatment with varying concentrations of DhL for 16 h, both adherent and floating cells were harvested and stained with Annexin V-FITC/PI according to the manufacturer’s procedure. The samples were analyzed with a BD FACSCanto II flow cytometer (Becton Dickinson).

### Western Blotting Analysis

Total protein extraction, quantification and immunoblots were performed as previously described [12]. Briefly, 20μg of total protein were separated by 12–15% SDS-PAGE and transferred to a PVDF membrane (IPVH00010, Immobilon-P, 0.45 μm, EMD/Millipore, Billerica, Boston, MA, U.S.A.). We used a mouse monoclonal antibody to CDKN1A (p21WAF1/Cip1, clone CP74, raised against the recombinant full-length human protein with N-terminal histidine tag, #05–655, Lot #2299569), a mouse monoclonal antibody to ß-actin (MAB1501R, Lot #LV1576007), a mouse monoclonal antibody to total TP53 (clone BP53-12, raised against a recombinant wild type tumor protein p53, #05–224, Lot #2326369), and a mouse monoclonal antibody against α-tubulin (clone AA2, #05–661, Lot #2207268), and all antibodies were obtained from EMD-Millipore. We also used a mouse monoclonal antibody to total TP53 (clone BP53-12, raised against a recombinant wild type tumor protein p53, sc-81168, Lot # C1413), a rabbit polyclonal antibody to phosphorylated p-S46-TP53 (raised against the phospho-Serine 46 peptide, sc-101764, Lot #G0313), a rabbit polyclonal antibody to total TP73 (recognizes all p73 isoforms raised against 1–80 amino acid peptide of human origin, sc-7957, Lot # D2413), a rabbit polyclonal antibody to p-Y99-TP73 (raised against the phosphor-Tyrosine 99 peptide, sc-101769, Lot # K2613), a rabbit polyclonal antibody to p-S139-histone **γ**H2A.X (raised against the phospho-Serine 139 peptide of human origin, sc-101696, Lot # F1814), and a rabbit polyclonal antibody to BAX (P-19, raised against a N-terminal epitope, sc-526, Lot # D0314), and all antibodies were obtained from Santa Cruz Biotechnology (Santa Cruz, CA, U.S.A.). All primary antibodies were used at the dilutions recommended by the manufacturer for immunoblotting. The appropriate goat anti-rabbit (AP307P) and goat anti-mouse (#AP308P, Lot #LV1560164) horseradish peroxidase-conjugated immunoglobulins (1:5000; EMD-Millipore) were subsequently used. Immunoreactive bands were visualized using an enhanced chemiluminescence kit with the SuperSignal West Pico Chemiluminescent Substrate (Pierce-Thermo Scientific, Rockford, IL, U.S.A.).

### Genotoxicity of Human Lymphocytes

The genotoxicity of DhL was assessed using two technical approaches. First we used the Cytokinesis-Block Micronucleus Assay (CBMN assay). Whole peripheral blood samples were obtained from three healthy donors (age range 20–25 years old). The donors were informed about the purpose of lymphocytes collection and signed a consent form. The heparinized peripheral blood (0.5 mL) was cultured for 72 h at 37°C in 6.3 mL of RPMI-1640 supplemented with 1% L-glutamine (Sigma-Aldrich, St. Louis, MO, U.S.A.), 1% non-essential amino acids (Sigma-Aldrich), and 0.2 mL phytohemaglutinin (Sigma-Aldrich). After 48 h of culture, cytochalasin B (Sigma-Aldrich) was first added at a final concentration of 6 μg/mL, and then three different concentrations of DhL, dissolved in DMSO, (Sigma-Aldrich) were tested (5, 15 and 25 μM). DMSO and mitomycin C (Sigma-Aldrich) were used as the solvent and positive controls, respectively. Aliquots of control and treated lymphocytes were removed for cell viability assay. The CBMN test was performed as reported elsewhere [13]. For each culture/concentration, 1000 binucleated cells were evaluated for associated micronuclei. Cell proliferation kinetics were analyzed by determining the frequency of mononucleated (Mono), binucleated (Bi) and polynucleated (Poly) cells, corresponding to 0, 1, and 2 or more in vitro cell divisions, respectively. Cytostatic activity was determined by calculating the nuclear proliferation index using the following equation: Nuclear Proliferation Index (NPI) = [#Mono +2#Bi + 3#Poly]/200 [13].

As a second genotoxicity test, we used a SCGE assay (comet assay). Heparinized blood samples (20 μL) were cultured at 37°C in RPMI 1640 medium (1.0 mL, Sigma-Aldrich) that was supplemented with L-glutamine (2 mM) and non-essential amino acids (10 mM, Gibco), and the samples were treated with various concentrations of DhL dissolved in DMSO (5–35 μM). DMSO and hydrogen peroxide (2.5 μM H_2_O_2_, Sigma-Aldrich) were used as the solvent and positive controls, respectively. The treated cells were incubated for 3 h at 37°C and then centrifuged. The supernatant was removed, and the pellets were washed with PBS and centrifuged again. The SCGE assay was performed as described elsewhere [14]. The DNA was allowed to unwind for 20 min in electrophoresis running buffer solution (300 mM NaOH, and 1 mM Na_2_EDTA, pH 13.0). Electrophoresis was conducted for 20 min at 25 V and 300 mA. Ethidium bromide (60 μL of a 1.5 μg/mL solution) was added to each slide, and a cover glass was placed on the gel; DNA migration was analyzed with a Zeiss Axioskop 2 plus 40 microscope with fluorescence equipment (filter 12) and measured with a scaled ocular. For the evaluation of DNA migration (tail length), 50 cells were scored for each plate.

### Statistical Analysis

All data were reported as the means ± SEM of independently performed experiments, as indicated in each figure. The statistical significance was obtained with one-way analysis of variance (ANOVA) followed by the Dunnet post-test. Treated cultures were compared to those observed in the ethanol control (GraphPad Prism 4). A p≤0.05 was considered to be statistically significant. The comet data were not normally distributed in our samples; therefore, we compared the group medians using the Mann-Whitney test and Kruskal-Wallis post-test to evaluate the differences between the compound concentrations and the control. Statistical analyses were carried out in GraphPad Prism 4 (GraphPad Software, San Diego, CA, U.S.A.).

## Results

### DhL Modulated Cell Viability of Cancer Cells

**Fig 1:**
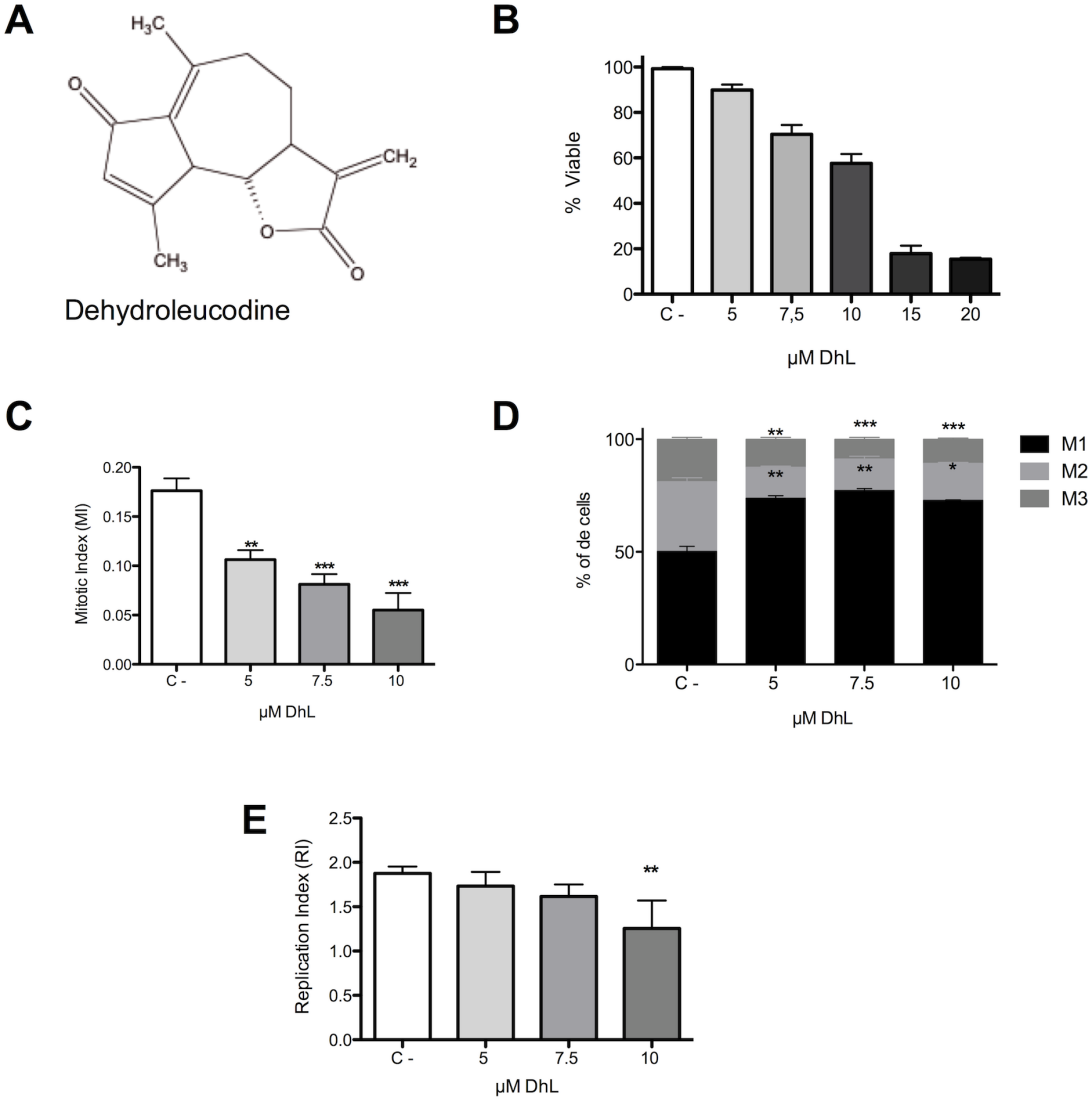
DhL treatment reduced the D384 cell survival. (A). The chemical structure of DhL (B). Analysis of D384 cell viability upon DhL exposure using a MTT assay after 16 h. The x-axis represents the varying concentrations of DhL (5–20 μM), and C- is the control sample without DhL. (C). D384 Mitotic index (MI) measurement upon DhL exposure (5–10 μM). (D). The fraction of D384 cells in 1 mitotic (M1), 2 mitotic (M2) and 3 mitotic (M3) stages after treatment with DhL (5–10 μM, C-, control, DMSO). (E). The replication index (RI) of D384 cells upon exposure to DhL (5–10 μM). Data represent mean ± SEM, n = 3. Symbols denote statistically significant differences: ** p<0.01, *** p< 0.001 with respect to (C-) control conditions (DMSO).

To determine whether DhL could affect tumor cell survival, we used a MTT assay to evaluate the inhibitory activity of the methanol extract of *G*. *verrucosa* on cell growth. Using four cancer cell lines (Table 1), we showed that the methanol extract of *G*. *verrucosa* more effectively inhibited the survival of cerebral astrocytoma D384 cells (Table 1). After subsequent purification of the methanol extract and spectroscopic analysis of the active compound, the sesquiterpene lactone DhL was identified (Fig 1A), and inhibited cell growth of all of the tumor cell lines tested, especially the human astrocytoma D384 cells (Table 1), after 48 h of exposure. D384 astrocytoma cells were selected for further studies.

**Table 1:** Effect of *G*. *verrucosa* methanol extract and DhL on the growth of human cancer cell lines.

Inhibition (% ± SE^a^)				
	Human cancer cell lines			
Treatment	D384 (Cerebral Astrocytoma)	A549 (Lung cancer)	MCF-7 (Breast cancer)	CAKI (Kidney cancer)
*Gynoxys verrocosa* 50 μg/mL	98.4 ± 1.3	79.3 ± 3.0	68.3 ± 6.3	90.0 ± 1.9
Dehydroleucodine 50 μM	98.9 ± 2.3	70.0 ± 2.9	69.1 ± 4.7	69.3 ± 4.5
Doxorubicin 1 μM	82.6 ± 1.2	74.5 ± 4.4	36.1 ± 3.1	47.8 ± 4.7

^a^ Standard error.

**Fig 2:**
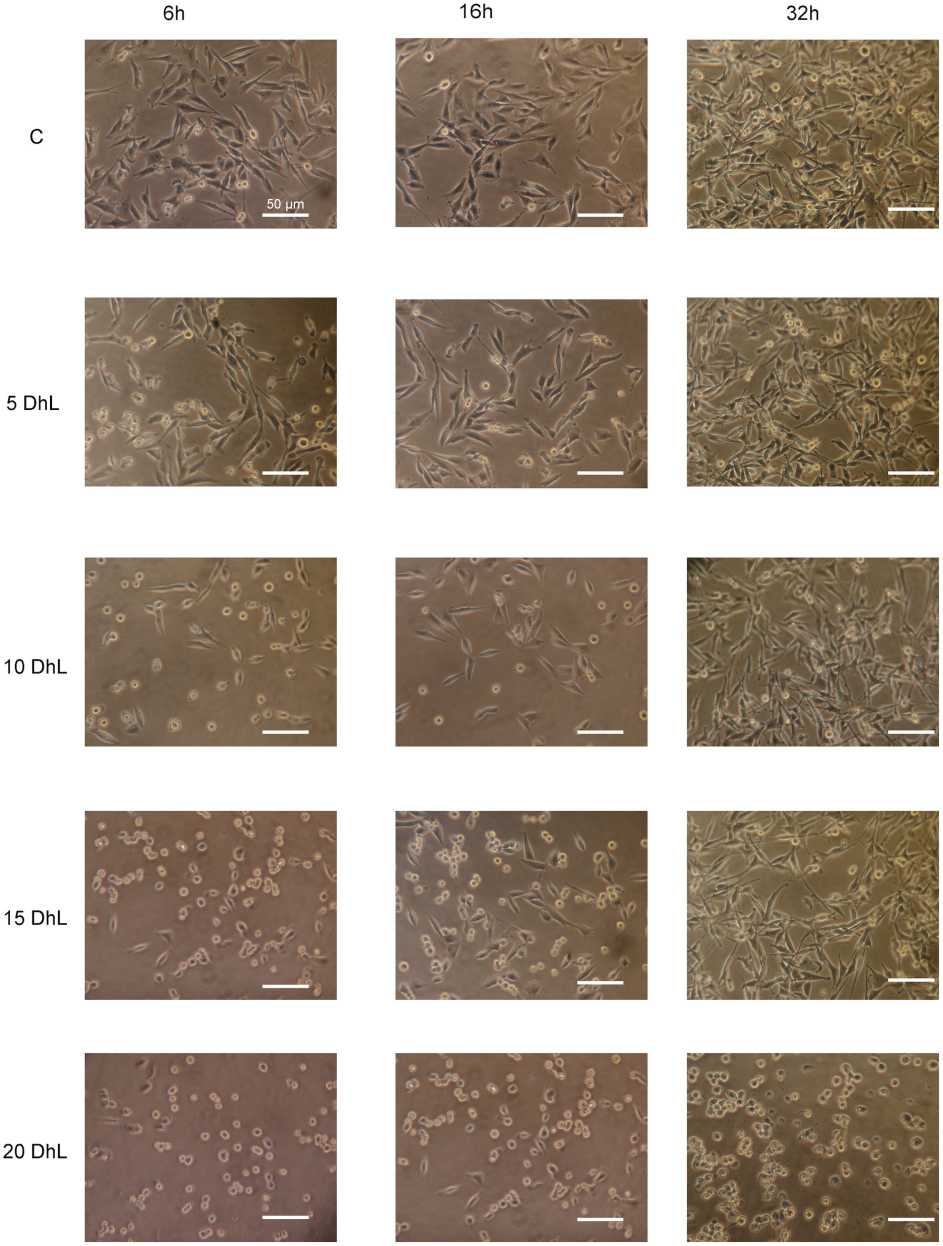
DhL modulated morphological changes in D384 cells. D384 cells were treated with 0, 5, 10, 15 and 20 μM DhL for 6 h, 16 h and 32 h, visualized under microscope and imaged. Magnification: 40X.

To examine the cancer cell viability upon DhL exposure, we used a MTT assay with D384 cells treated with the various concentrations of DhL (5, 7.5, 10, 15 and 20 μM) for 16 h (after a replication cycle). We found that the cancer cells were viable up to 10 μM, whereas concentrations of 15 μM and greater resulted in a dramatic decrease in the viability of D384 cells (up to ~40%, Fig 1B). When D384 cells were exposed to various concentrations of DhL for 16 h, morphological changes were observed (Fig 2). The cancer cells treated with the negative control showed the characteristic morphology of the cell line and ~60% density. However, at the 5 μM and 7.5 μM concentrations, a decrease in density was observed, while the cell morphology remained intact. Morphological changes were more obvious at the 10 μM and 15 μM doses. When the cells were exposed to 2 rounds of replication, the density control was ~80%, while the cytotoxic effects were accentuated at 15 μM. These observations, as well as the cell viability data, suggest that DhL displayed distinct effects depending on the concentration used (cytostatic at low concentration and cytotoxic at high concentration).

As shown in Fig 1C, the mitotic index of D384 cells decreased after treatment with DhL in a concentration-dependent manner compared to the negative control (with an index of 0.176, which decreased significantly to 0.055 at the highest tested concentration of 10 μM, p < 0.0001). Using a BrdU incorporation assay, we differentiated distinct metaphase division cycles (Fig 1C) and showed that, at the tested concentrations, DhL decreased the fraction of cells in the second and third stages of mitosis, while the amount of cells in the first stage of mitosis was increased (compared to the control), at all concentrations tested (Fig 1D). This effect was confirmed by establishing the rate of replication (Fig 1E), showing that at a concentration of 10 μM, RI is 1.255, which decreased compared to the rate obtained for the negative control (1.875, p <0.001).

### DhL Displayed a Cytostatic Effect in D384 Cells through Cell Cycle Arrest and Apoptosis

**Fig 3:**
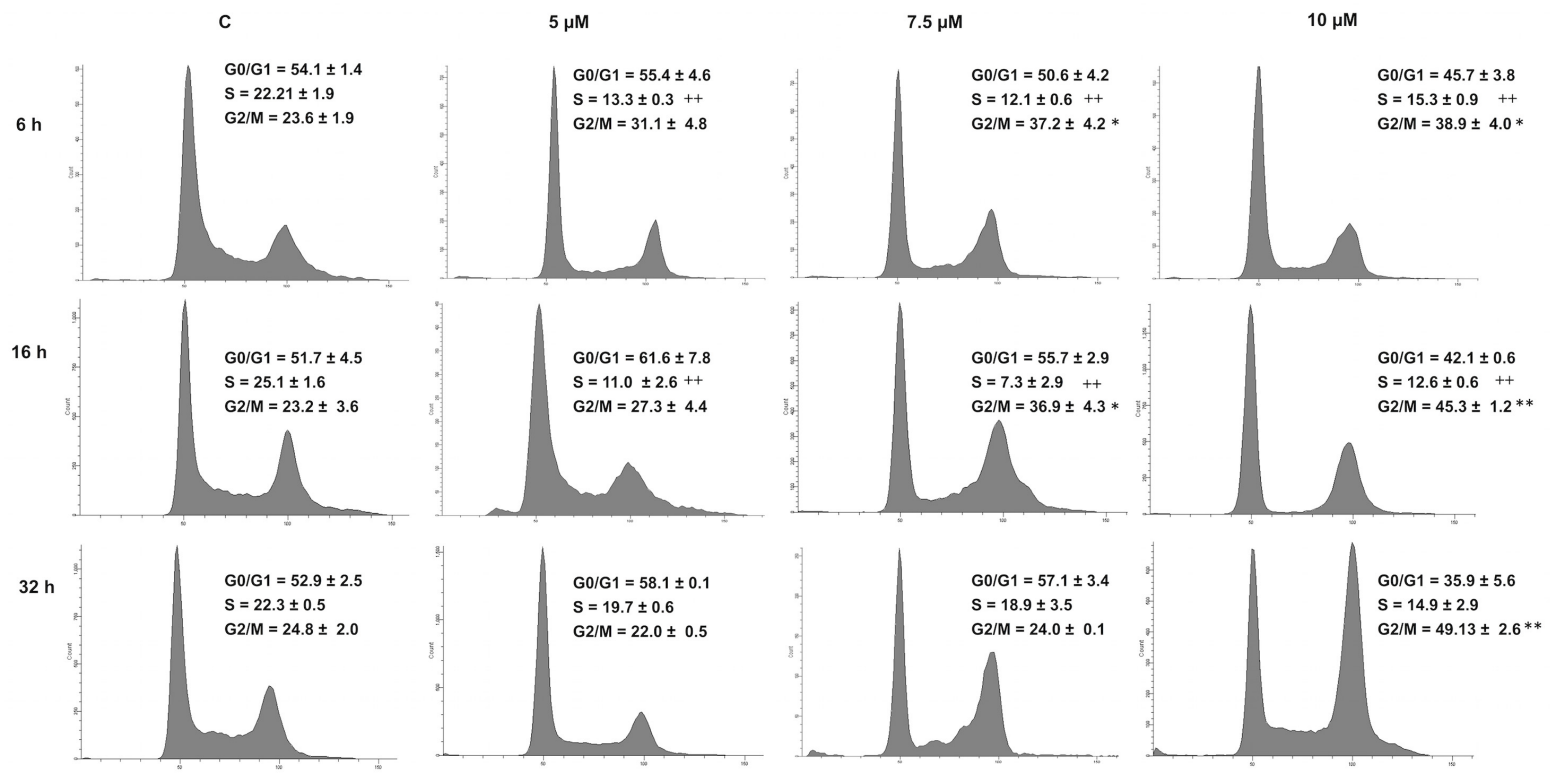
DhL induced cell cycle arrest in D384 cells. Cell cycle analysis. D384 cells were exposed to 0 (C-, control), 5, 7.5 and 10 μM of DhL for 6, 16 and 32 h. The fraction of cells in the G1, S, and G2/M-phases of the cell cycle was then analyzed using a FACSCanto II flow cytometer. Data were acquired and analyzed using DIVA software. A representative histogram is shown for each DhL concentration. Data represent the mean ± SEM, n = 4. Symbols denote statistically significant differences: * p<0.01 and ** p<0.001 with respect to control conditions for G2/M phase and ++ p< 0.001 with respect to control conditions for S phase.

We tested the effect of DhL on the cell cycle distribution using the same concentrations (5 μM, 7.5 μM and 10 μM) and varying times (6, 16 and 32 h). We showed that the exposure of D384 cells to DhL for 6 h and 16 h induced a significant decrease of the number of D384 cells in S phase compared to the control (p <0.0001) and was accompanied by an increase in the fraction of cells in the G2/M phase at all concentrations (Fig 3). Although the exposure of D384 cells to DhL concentrations of 5 μM and 7.5 μM for 32 h showed no change with respect to the control, the treatment of cells with 10 μM of DhL for 6 h and 12 h showed a decrease of a number of cells in the G0/G1 phase accompanied by an increase in the number of cells in the G2/M phase.

**Fig 4:**
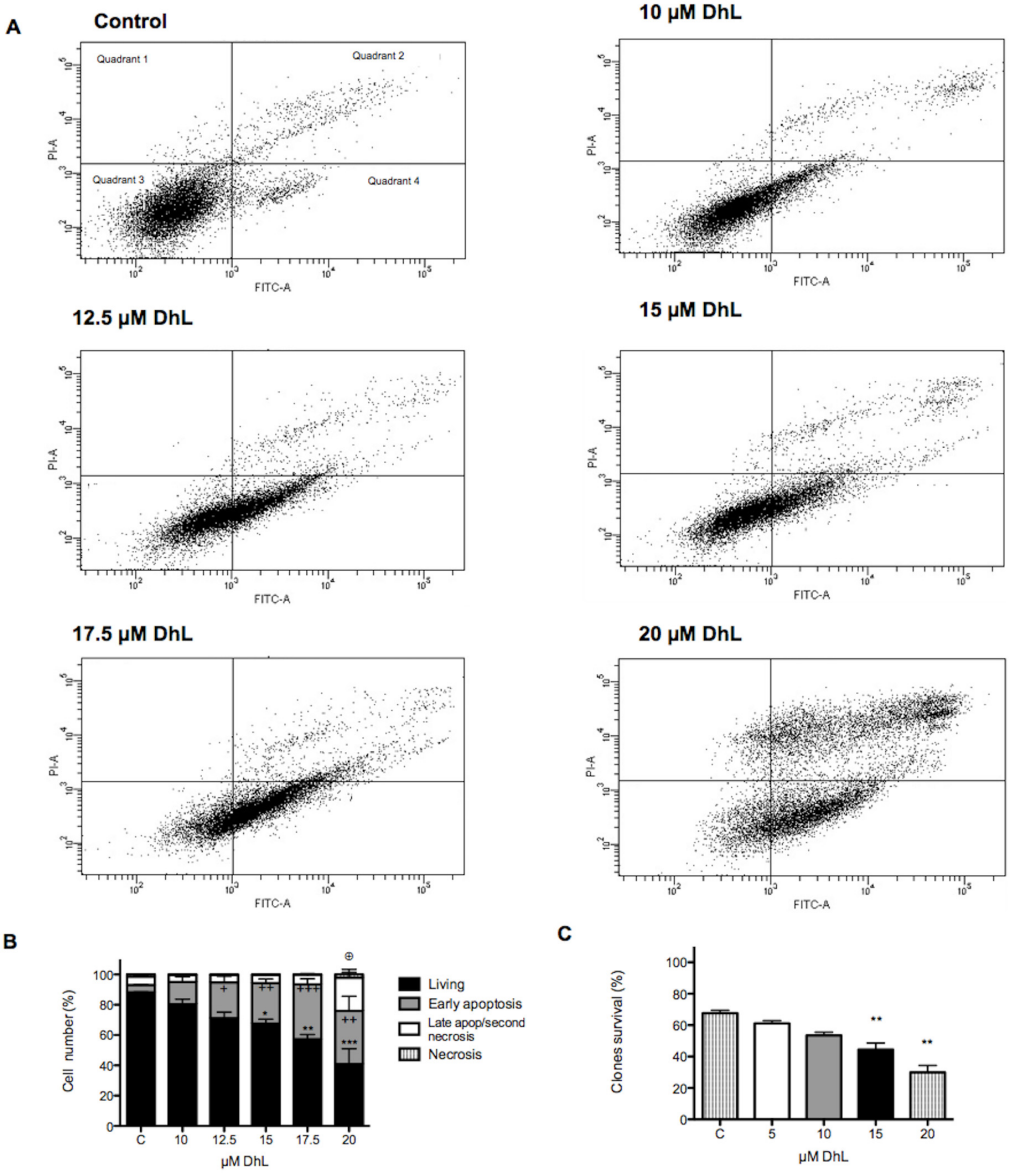
Apoptotic and clonogenic effect of DhL on D384 cells. Apoptotic D384 cells were detected and monitored by Annexin V-FITC/PI staining. (A) A representative histogram is shown for each DhL concentration. Quadrant 1, PI (+) (cells undergoing necrosis); Quadrant 2, Annexin V-FITC (+) and PI (+) (cells in the late apoptosis and secondary necrosis); Quadrant 3, Annexin V-FITC (−) and PI (−) (living cells); Quadrant 4, Annexin V-FITC (+) and PI (−) (cells in the early apoptosis). (B) Apoptotic changes. The fractions of live (black bars), early apoptotic (gray bars), late apoptotic/secondary necrotic (white bars) and necrotic (striped bars) cells are shown. Symbols denote statistically significant differences: * p<0.01, ** p<0.001 and *** p<0.0001 with respect to control conditions for live cells, +p< 0.01, ++p< 0.001 and +++p< 0.0001 with respect to control conditions for early apoptosis, and ⊕ p<0.01 with respect to control conditions for late apoptosis/secondary necrosis. (C) Clonogenic assay. Cells were treated with DhL for 16 h. The number of counted colonies was expressed as a fraction of the control (defined as 100%). Symbols denote statistically significant differences: * p< 0.01, ** p<0.001 vs. control. Data are represented as the mean ± SEM (n = 4) of 3 independent experiments.

We further examined the cytotoxic effect of DhL at the higher concentrations (10, 12.5, 15, 17.5 and 20 μM). Fig 4A–4F shows the results of flow cytometry analysis of D384 cells using the Annexin V-IP assay. We observed that increasing concentrations of DhL induced the D384 cell migration from the quadrant of living cells to the quadrants of early apoptotic cells and late apoptotic cells. The fraction of living cells in the control group was 88%, whereas 40.8% of the cells survived at the higher concentration of DhL (Fig 4G). As shown, the rate of the early apoptotic cells in control sample was ~4.8%, which significantly increased after treatment with 17.5 μM DhL (~38.4%) or 20 μM DhL (~33.3%). Simultaneously, the rate of late apoptotic D384 cells exposed to 20 μM DhL (19.5%) also increased when compared with the control cells (5.5%) (Fig 4H). Apparently the cytostatic effect was temporary because the removal of DhL resulted in the reversion of D384 cell viability to > 80% at a concentration of 10 μM. Treatment with higher concentrations of DhL maintained the cytotoxic effect; the cell survival of D384 cells did not exceeded ~44% compared to control (Fig 4H).

### DhL Induced the Expression of Total p73 and Phosphorylation of p73 and p53 in D384 Cells

**Fig 5:**
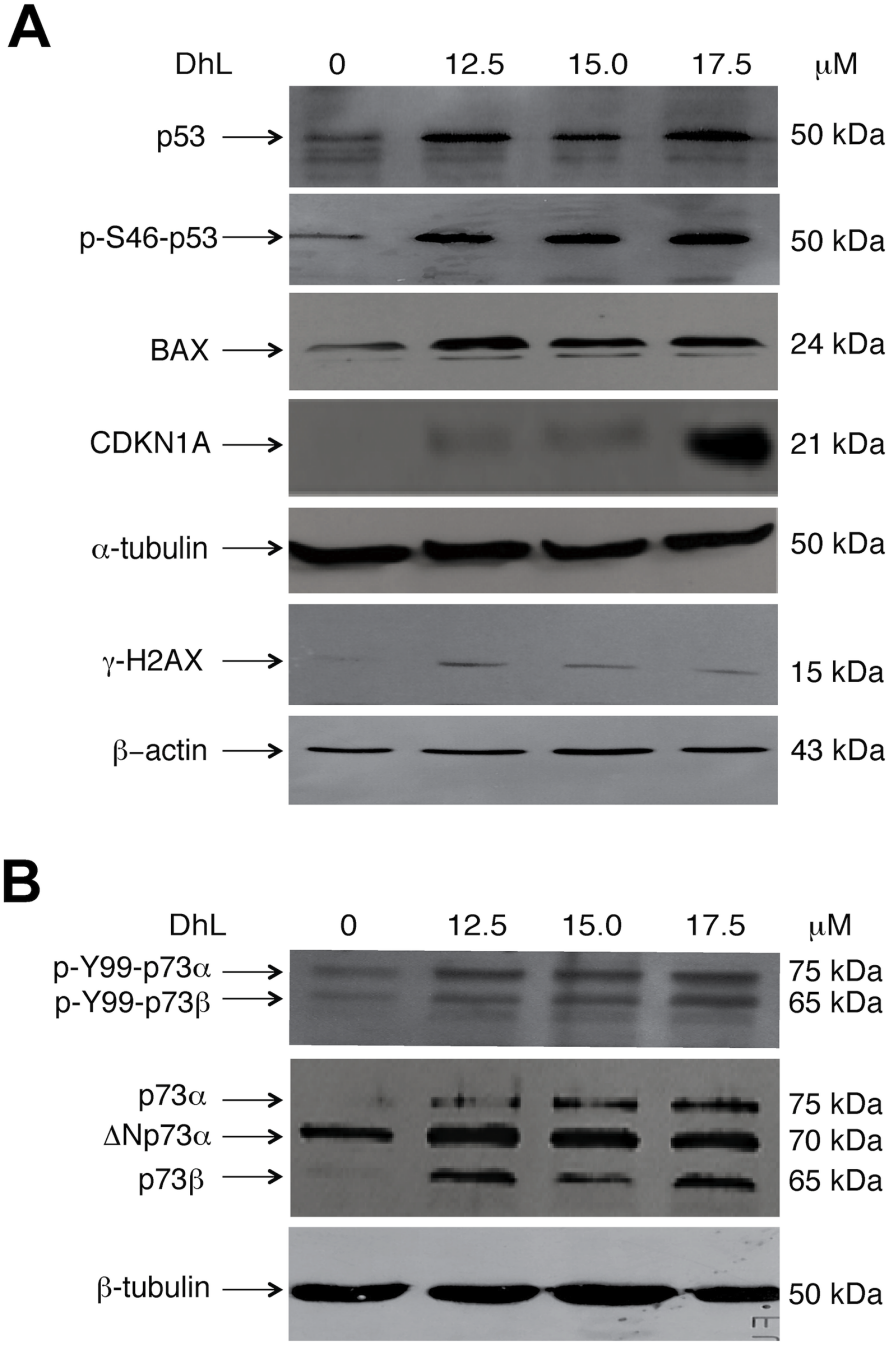
DhL induced the expression of proteins involved in cell cycle arrest and the apoptotic response of D384 cells. D384 cells were exposed to 12.5, 15 and 17.5 μM DhL for 16 h. Total protein lysates were separated by 12–15% SDS-PAGE followed by western blot analysis with the indicated antibodies against BAX, CDKN1A, TP53, S46-p-TP53, and p139-**γ**H2AX, as shown in Panel A, and TP73 and Y99-p-TP73, as shown in Panel B. **α**-Tubulin and ß-actin were used as loading controls for total cell extracts.

Accumulating evidence shows that the p53 family members (p53, p63 and p73) play a fundamental role in the regulation of cell cycle arrest, apoptosis, autophagy and metabolism in cancer cells exposed to stress-inducing and DNA damaging agents of various origins [15, 16]. Therefore, we examined whether DhL-induced apoptotic cell death involved the expression of these transcriptional factors in D384 cells. The notion of the involvement of TP53 family members in the response of cancer cells to DhL was also supported by the initial observations that DhL induced the expression of the cell cycle regulator, CKND1A (p21), a well-known downstream target of all TP53 family members [15, 16]. Using the total lysates obtained from D384 cells treated with the control and increasing concentrations of DhL (12.5, 15 and 17.5 μM for 16 h), we found that the expression of CDKN1A (p21) increased upon DhL exposure in a concentration-dependent manner (Fig 5). We further found that another downstream target of the TP53 family, the apoptotic activator BAX, was also up regulated in D384 cells upon DhL exposure (Fig 5). The increased levels of the proapoptotic BAX protein supports the apoptotic mechanism implicated in the DhL response, whereas the increase in phosphorylated levels of **γ**-H2AX (p139-H2AX) suggests the genotoxic effect of DhL in D384 cells.

We next observed that while the expression of total TP53 was not changed in D384 cells treated with increasing concentrations of DhL, the total level of TP73 was increased (Fig 5). Moreover, we also showed that levels of phosphorylated S46-p-TP53 and Y99-p-TP73 were also increased upon the D384 astrocytoma cell response to DhL (Fig 5). These observations supported the hypothesis that the death observed in D384 cells upon DhL exposure is likely regulated by p73 and perhaps through S46-p-p53 and Y99-p-p73 as well.

### DhL Displayed Cytostatic/Genotoxic Activities in Human Lymphocytes

**Fig 6:**
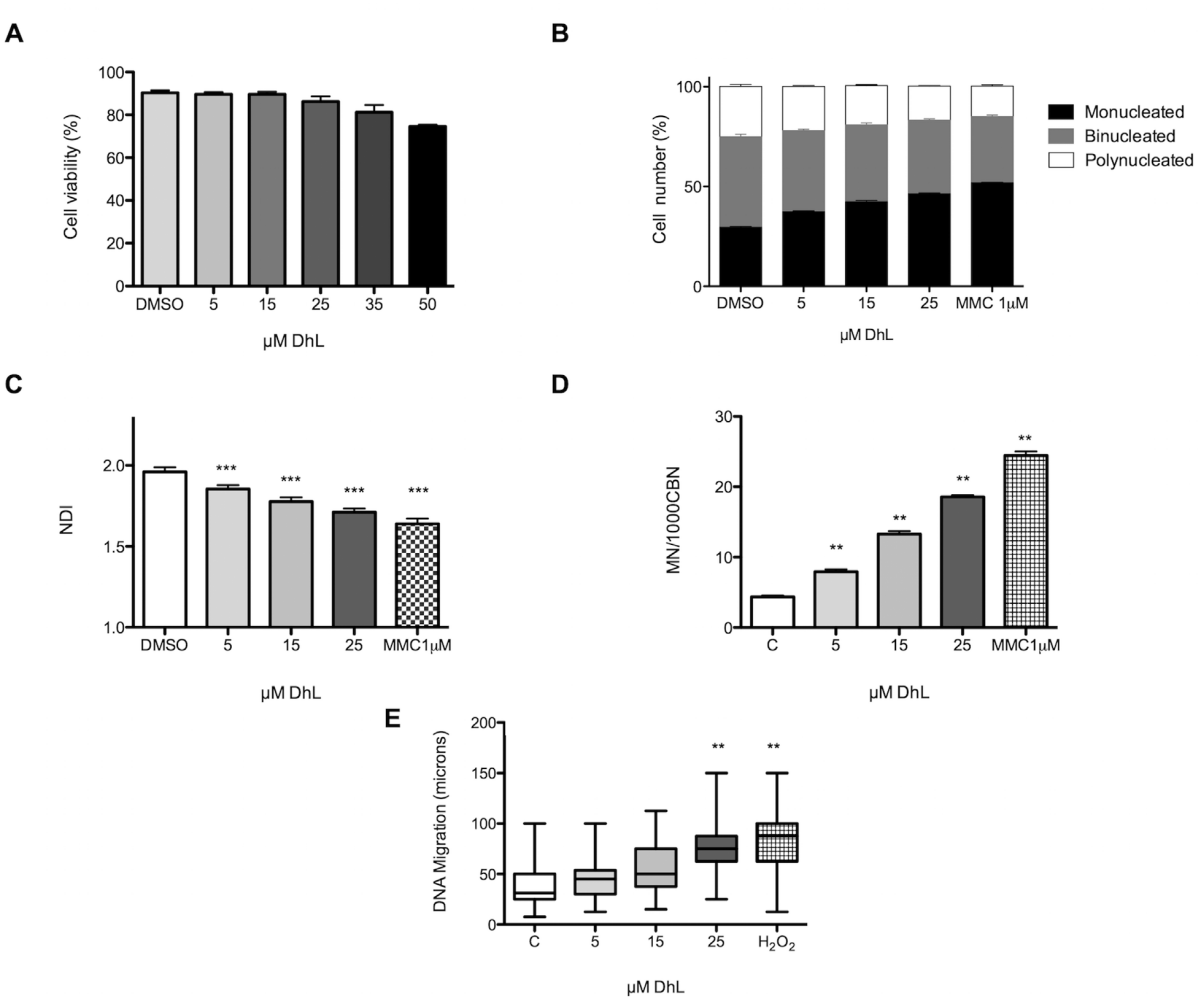
DhL induced cytostatic and genotoxicity effects in human lymphocytes. Lymphocytes were exposed to DhL (5, 15, 25, 35 and 50 μM). (A) Cell viability assay. (B) Proliferation kinetics of lymphocytes. (C) Nuclear division index (NDI) assay. The fraction of monucleated (black bars), binucleated (grey bars) and polynucleated (white bars) cells are shown. ANOVA-Dunnet: * p <0.005, ** p<0.001, *** p <0.001. (D) Micronucleus frequency (MN) on binucleated cells. As a reference, we used lymphocytes treated with 1 μM Mitomycin C (MMC) (square bars) ANOVA-Dunnet: **p <0.001. (E) Comet assay. The tests for significance were limited to the Kruskal–Wallis one-way ANOVA-Bonferroni on ranks. (** p <0.001). The data are represented as the mean (±SEM) of three duplicated experiments from three donors.

As with all sesquiterpene lactones, DhL can interact with DNA and proteins [17], leading us to further examine the potential genotoxic effects of DhL in control lymphocytes using in vitro genotoxicity assays (CBMN assay and comet assay). To establish working subtoxic concentrations for genotoxicity testing, the cytotoxic effect of DhL on human lymphocyte proliferation was assessed with fluorescein diacetate (FDA)/ethidium bromide staining. Lymphocytes were exposed to DhL for 24 h and showed no decrease in viability at any of the concentrations tested, unlike cancer cells (Fig 6A). We assessed the effect of DhL treatment on the kinetics of lymphocyte cell proliferation (CPK) and calculated the nuclear division index (NDI) using both parameters (CPK and NDI) as cytostatic criteria for human lymphocyte proliferation. The effects of DhL on cell proliferation were evaluated by counting the proportion of monucleated, binucleated, and polynucleated cells. Exposure of lymphocytes to DhL for 24 h resulted in an increased fraction of mononuclear cells, while the fraction of multinucleated cells decreased (Fig 6B). Furthermore, DhL decreased the proliferation of lymphocytes as assessed by NDI, suggesting that DhL acts as a cytostatic agent in normal lymphocytes (Fig 6C).

When we assessed lymphocyte proliferation in binucleate cells as described elsewhere [13], where the genotoxic effect was determined based on the frequency of micronuclei, we observed that DhL (starting from 5 μM) increased the micronuclear frequency (Fig 6D). In the comet assay, lymphocytes were exposed to various concentrations of DhL for 3 h and the length of the “comet” tail was measured (Fig 6E). With this assay, we observed that the exposure of lymphocytes to DhL statistically increased the migration of the comet tail at 25 μM (Fig 6E).

## Discussion

Historically, natural products from plants and animals were the source of virtually all medicinal preparations and, more recently, natural products have continued to enter clinical trials or to provide leads for compounds that have entered clinical trials, particularly as anticancer [18–21]. The search for anticancer drugs has been governed by the fact that cancer cells replicate more rapidly than normal cells, and the vast majority of the currently used drugs cause DNA damage, thereby interrupting cell division and subsequently causing cell death [22, 23]. Bioactive phytometabolites with lesser toxic effects are widely available in the natural habitats of many countries, especially in the diverse Amazonian flora in South America [23]. These phytometabolites, derived from plants, fungi, marine organisms, modulate multiple molecular targets and affect a number of signaling and regulatory pathways, which ultimately leads to tumor cell death via cell cycle arrest, apoptosis or necrosis [24–43]. Phytometabolites are functionally pleiotropic and may affect multiple intracellular targets and different cell signaling processes that are usually altered in cancer cells with limited toxicity in normal cells [24, 33]. The simultaneous targeting of multiple pathways may help to kill cancer cells and slow the onset of drug resistance [38, 39, 44].

Several preclinical investigations have shown that certain natural phytometabolites, such as curcumin, resveratrol, timosaponin III, gallic acid, diosgenin, pomegranate, epigallocatechin gallate, genistein and 3,3'-diindolylmethane, inhibit the mTOR pathway directly or indirectly [43]. D-pinitol is a naturally occurring compound derived from soy that significantly inhibited the proliferation of MCF-7 cells in a concentration-dependent manner. These phenotypic changes coincided with the increased expression of TP53 and BAX, as well as the down regulation of BCL2 and NF-κB, thereby inducing cell death through an apoptotic mechanism [45]. The plant-derived berberines are shown to be cytotoxic to human colon cancer cells and are more effective on cells harboring wild type TP53 in which they promote cell cycle arrest and DNA damage, as well as trigger caspase-dependent apoptosis and drive autophagy [46]. The DNA topoisomerase I inhibitor β-lapachone is one of the phytometabolites that induces a cell-cycle delay at G1/S phase before inducing either apoptotic or necrotic cell death in a variety of human carcinoma cancers [47].

The current study describes the ability of the phytometabolite DhL, from the species *G*. *verrucosa* that is grown in the certain areas of South Ecuador, to inhibit tumor cell growth *in vitro*. Among the four tumor cell lines tested, we observed that the most sensitive to DhL exposure was the D384 astrocytoma cell line. We found that both extracts from *G*. *verrucosa* biomass and purified DhL negatively affected the survival of D384 cells *in vitro*. Additionally, we found that DhL could induce both cell cycle arrest and apoptosis in D384 astrocytoma cells in a concentration-dependent manner. We further found that the concentrations at which DhL was cytotoxic to D384 cancer cells did not harm normal human lymphocytes. At low concentrations, DhL induced a cytostatic effect, similar to that reported for sesquiterpene lactones such as Parthenolide, Deoxyelephantopin, Isodeoxyelephantopin and Costunolide [48–51]. This effect was also consistent with the anti-proliferative effects reported for DhL and 11,13-dihydro-dehydroleucodine on vascular smooth muscle cells, as well as Dehydroparishin-B on melanoma B16 cells [52, 53]. At high concentrations, the apoptotic effect of DhL on D384 cells was found to be similar to that observed in MCF-7 breast cancer cells and HeLa cervical-uterine cancer cells [54].

The molecular mechanisms that regulate the cell cycle and apoptosis are closely related; thus, a number of proteins that control cell cycle progression may also induce apoptosis under conditions in which cell cycle progression is not adequately developed [55, 56]. We observed that the DhL-induced cell death phenotype is associated with the increasing expression of the transcription factor TP73, the apoptotic activator BAX and the cell cycle inhibitor CDKN1A (p21), as well as with the increasing phosphorylation of TP53 and TP73 at the S46 and Y99 amino acid residue, respectively. Finally, we found that DhL induced the phosphorylation of the DNA damage marker, **γ**-H2AX (p-S139-H2AX).

The latter observation that specific post-translational modifications of TP53 and TP73 in D384 cells upon DhL exposure strengthens the idea that DhL induces apoptotic machinery, because these phosphorylation events are often associated with the pro-apoptotic activities of TP53 and TP73 [57–62]. c-ABL phosphorylates TAp73α on tyrosine Y99, which is a necessary step for the pro-apoptotic activity of TP73 and its ability to transcriptionally activate BAX. This phosphorylation subsequently leads to the translocation of BAX to the mitochondria to induce cytochrome c release from the mitochondria [57–59].

Many chemotherapeutic drugs induce the expression of TP73, which in turn enhances drug-induced TP73-dependent apoptosis. Several studies have shown the involvement of TP73 in response to phytometabites [63]. 4-(39,39-dimethylallyloxy)-5-methyl-6-methoxyphthalide (MP) was obtained from the liquid culture of *Pestalotiopsis photiniae* isolated from the Chinese Podocarpaceae plant *Podocarpus macrophyllus*. Flow cytometry showed that MP induced G1 cell cycle arrest and apoptosis, as well as apoptotic morphology of cancer cells in a concentration-dependent manner [63]. The expression of the TP73 protein was increased after treatment with various MP concentrations [63]. The cell cycle-related genes, CDKN1A, CDKN2A and GADD45A, and the apoptotic regulator BIM, were significantly upregulated upon MP exposure [63].

The tumor cell death phenotype induced by DhL could largely be related to DNA damage, as indicated by micronuclei and comet assays. The increase in CDKN1A and **γ**-H2AX expression in D384 cells upon DhL exposure also indicated cell cycle arrest and DNA damage response of cancer cells, in which DhL would induce DNA repair mechanisms. Because of the potential failure of DNA repair machinery, the cancer cells are forced to enter the process of apoptotic cell death as shown by the increasing expression of BAX, which has been reported in other studies [20]. Overall, our results support the idea that *G*. *verrucosa* extract and purified DhL can inhibit the growth of the tumor lines, particularly of D384 astrocytoma cells, by activating cell cycle arrest and apoptosis through a TP73-dependent mechanism. The active substance, DhL, was shown to display both cytostatic and cytotoxic activities, as well as the genotoxic effects, in D384 cells in a concentration-dependent manner. The molecular events underlying these effects include, but are not limited to, TP73, p-TP73, p-TP53, CDKN1A, and BAX-dependent pathways. Further in-depth studies are needed to pinpoint other numerous players involved in the effects of DhL to fully understand the molecular landscape underlying the response of cancer cells to phytometabolite DhL.

## Supporting Information

S1 Fig NMRs Spectrum correspond to the dehydroleucodine. (PDF)
