# Correction: Phytometabolite Dehydroleucodine Induces Cell Cycle Arrest, Apoptosis, and DNA Damage in Human Astrocytoma Cells through p73/p53 Regulation

**DOI:** 10.1371/journal.pone.0173648

**Published:** 2017-03-09

**Authors:** Natalia Bailon-Moscoso, Gabriela González-Arévalo, Gabriela Velásquez-Rojas, Omar Malagon, Giovanni Vidari, Alejandro Zentella-Dehesa, Edward A. Ratovitski, Patricia Ostrosky-Wegman

There is an error in the first sentence of the “Cell Lines” paragraph of the Materials and Methods section. Dr. Uliana de Simone did not provide cell lines for this study. The correct sentence is: Human astrocytoma D384 cells were provided to the authors as a gift.
