# Supplementary material for: Phytometabolite Dehydroleucodine Induces Cell Cycle Arrest, Apoptosis, and DNA Damage in Human Astrocytoma Cells through p73/p53 Regulation
Source: PLoS One. 2015 Aug 26;10(8):e0136527. doi: 10.1371/journal.pone.0136527 (PMC4550445; doi:10.1371/journal.pone.0136527)
Supplement: S1 Fig — (PDF) [file pone.0136527.s001.pdf]

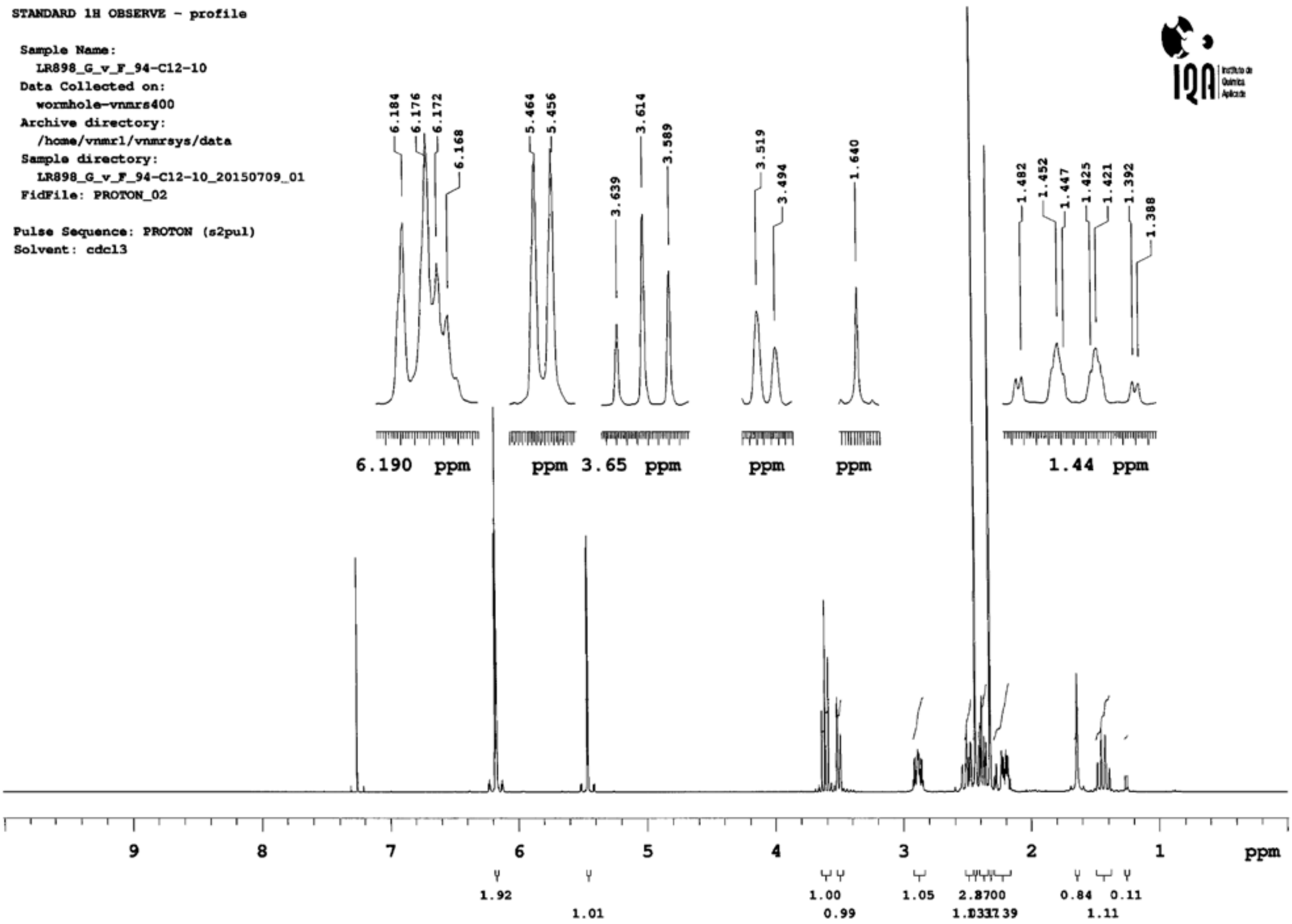

^{1}H NMR spectrum of DhL in CDCl3

STANDARD 1H OBSERVE - profile

Sample Name:
LR898_G_v_F_94-C12-10
Data Collected on:
wormhole-vnmrs400
Archive directory:
/home/vnmr1/vnmrsys/data
Sample directory:
LR898_G_v_F_94-C12-10_20150709_01
FidFile: PROTON_02

Pulse Sequence: PROTON (s2pul)
Solvent: cdcl3

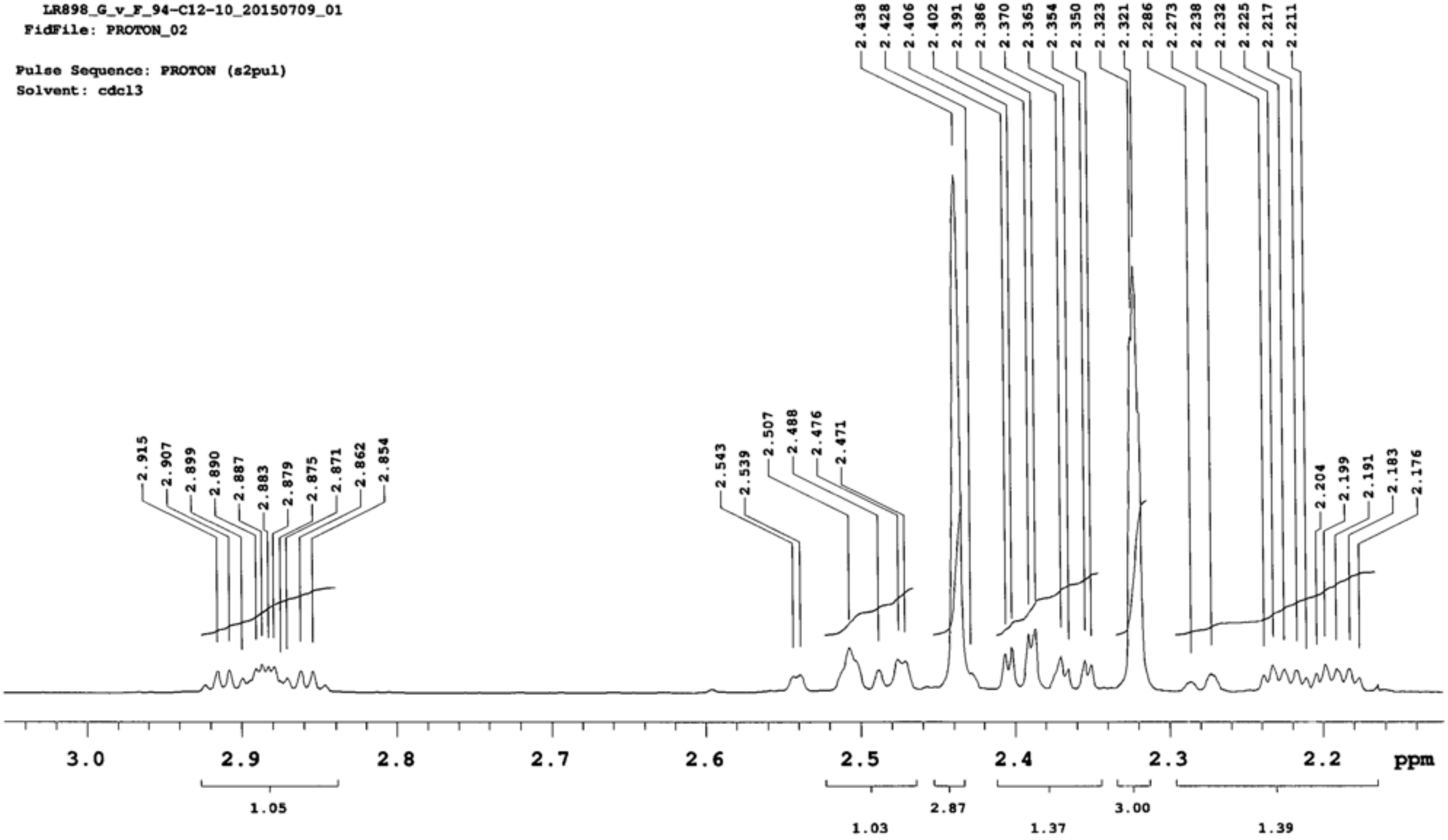

^{1}H NMR spectrum of DhL in CDCl3

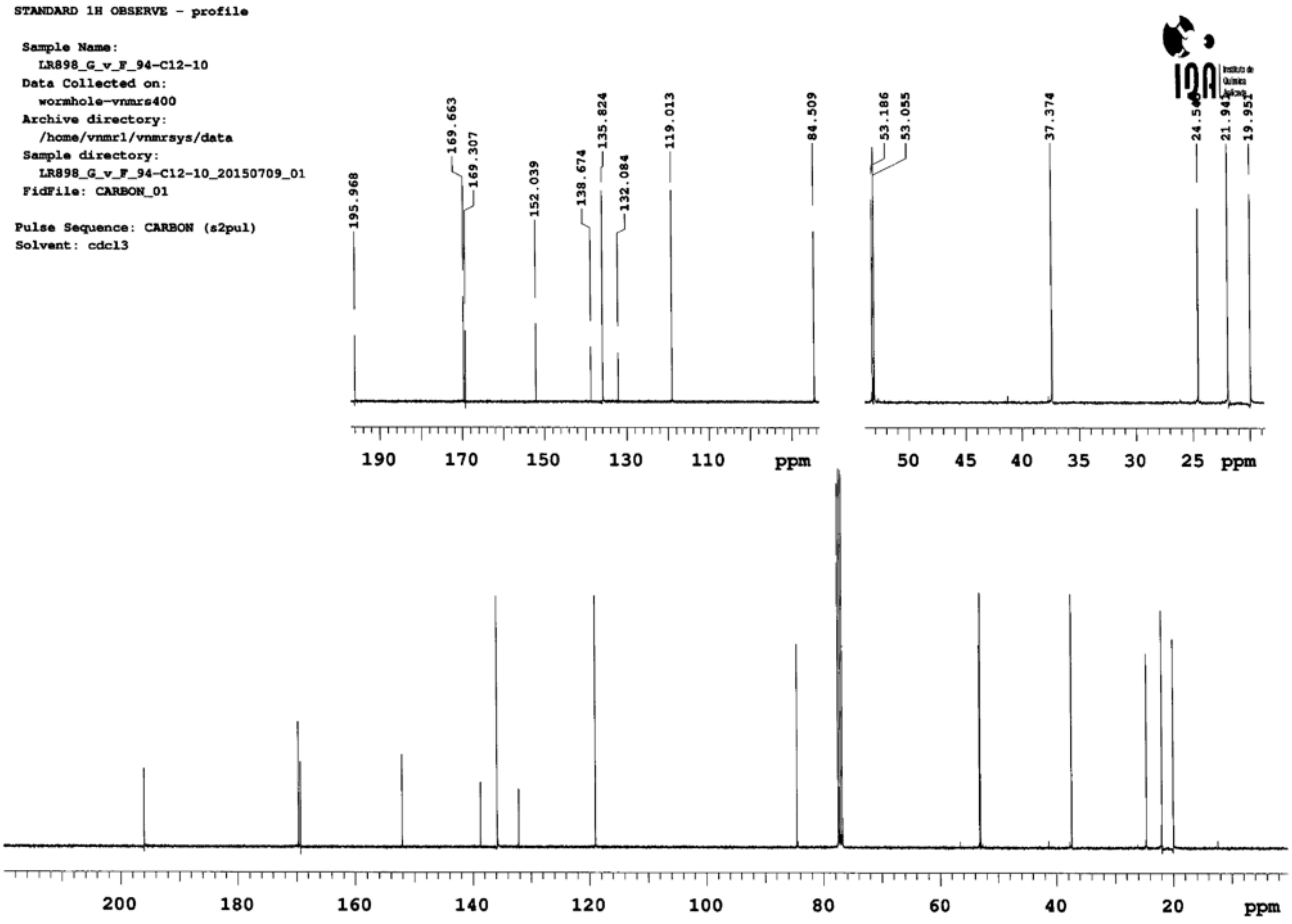

^{13}C NMR spectrum of DhL in CDCl3
